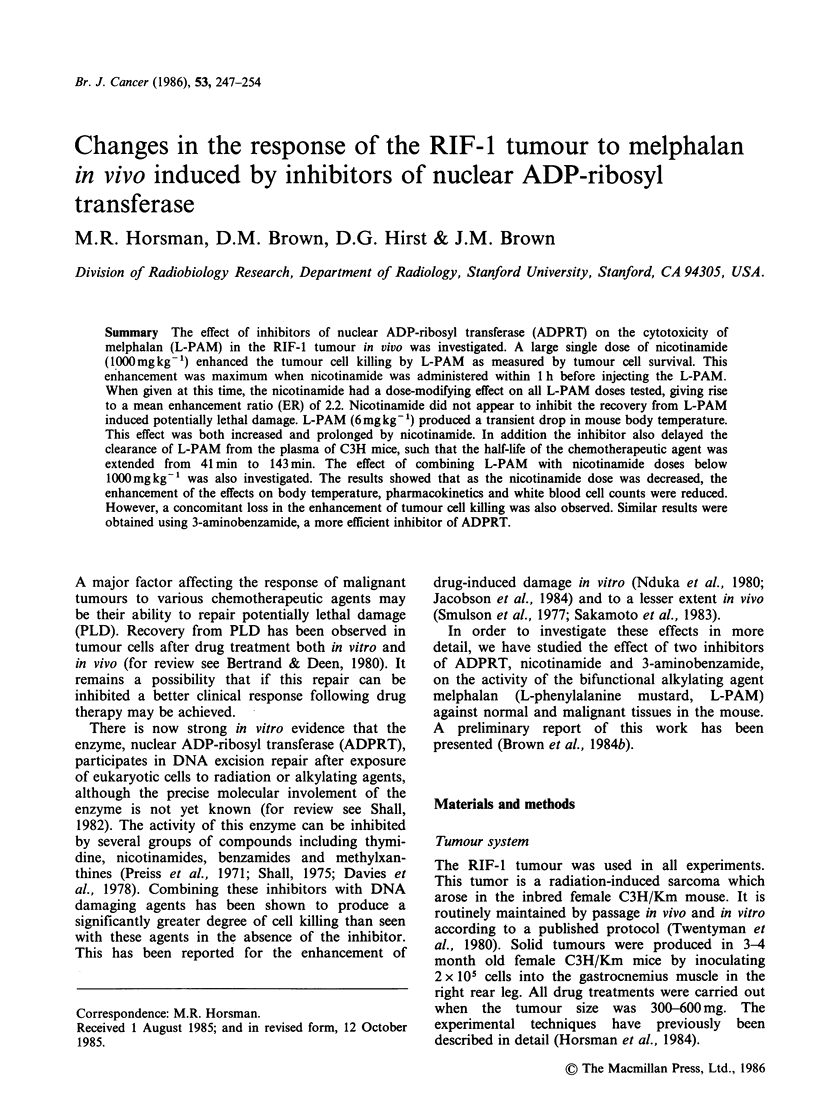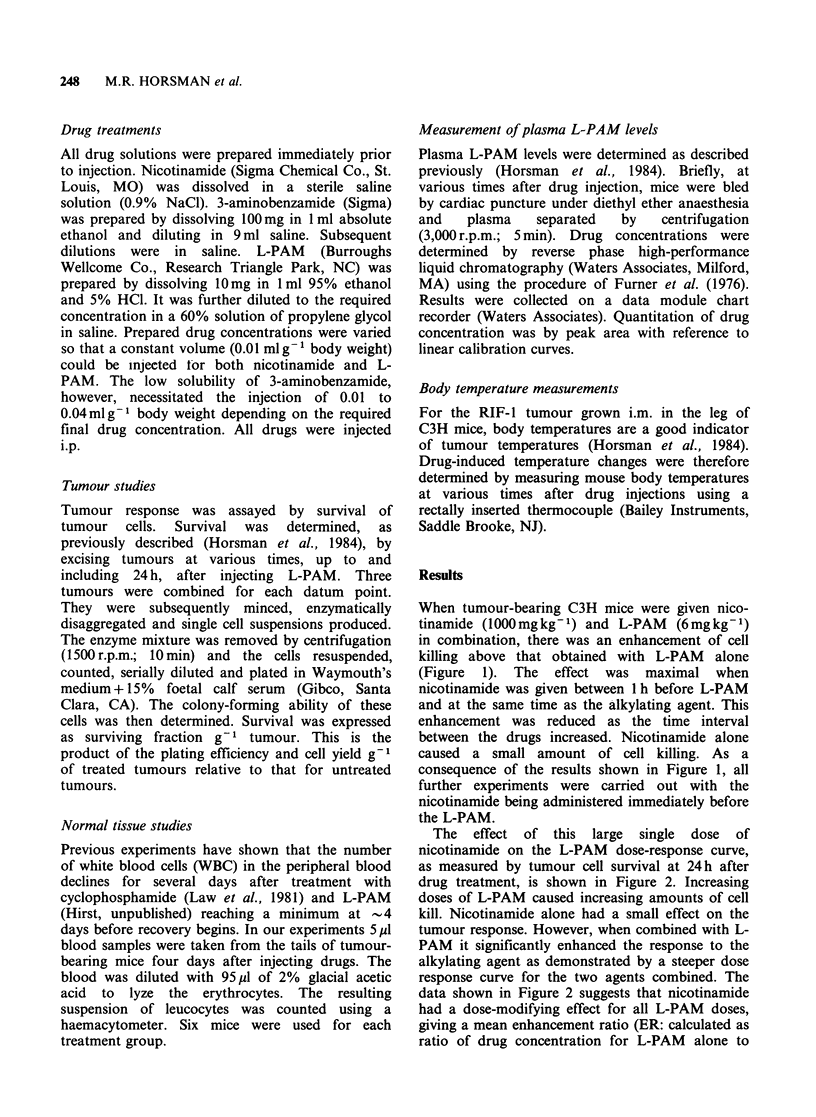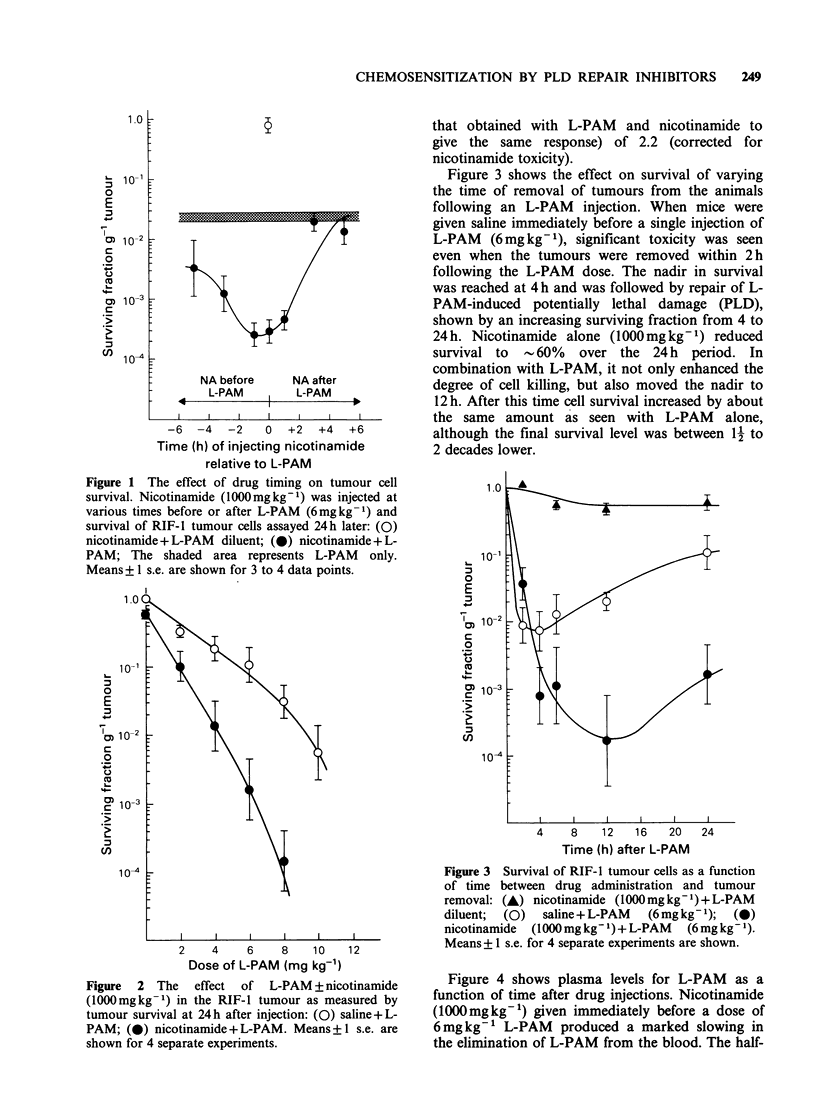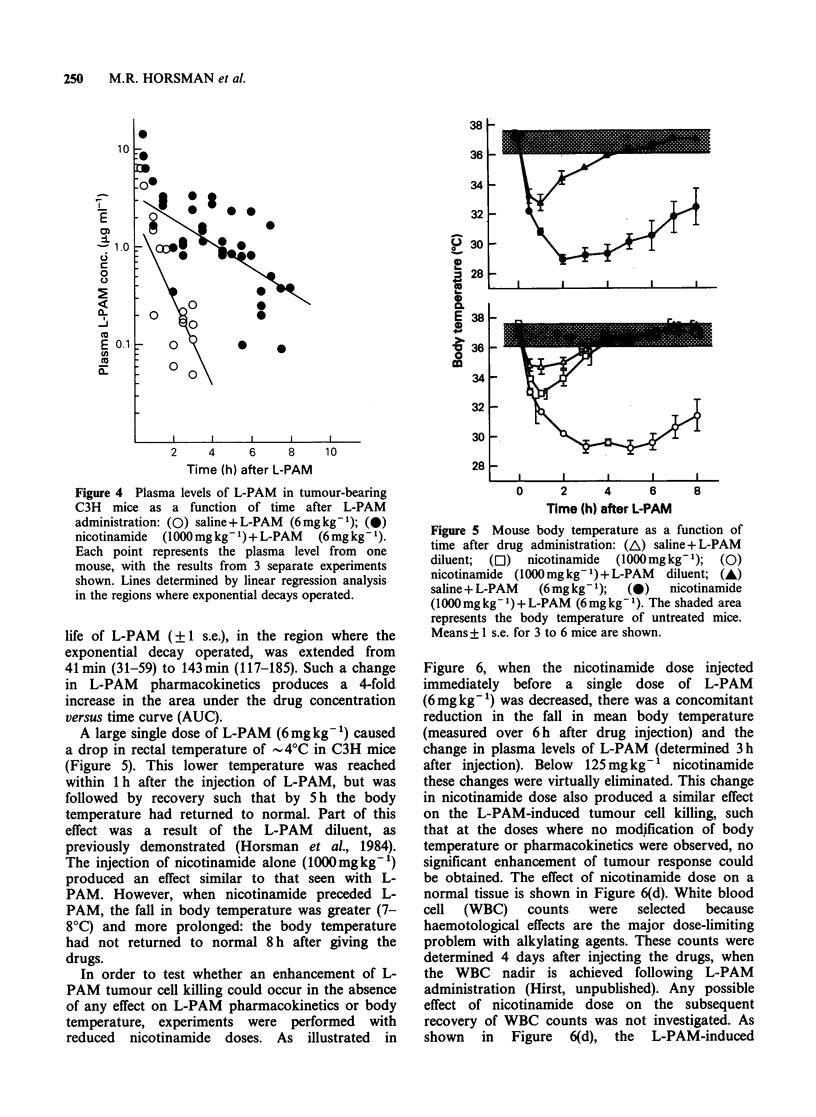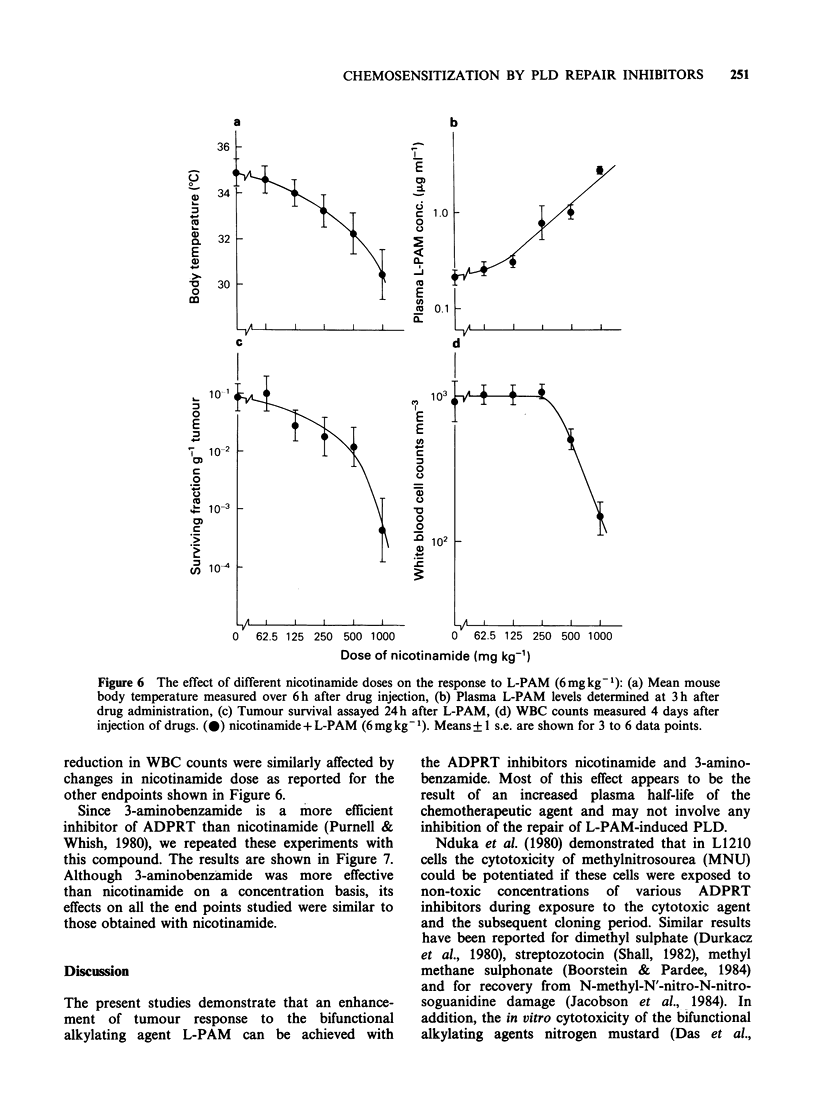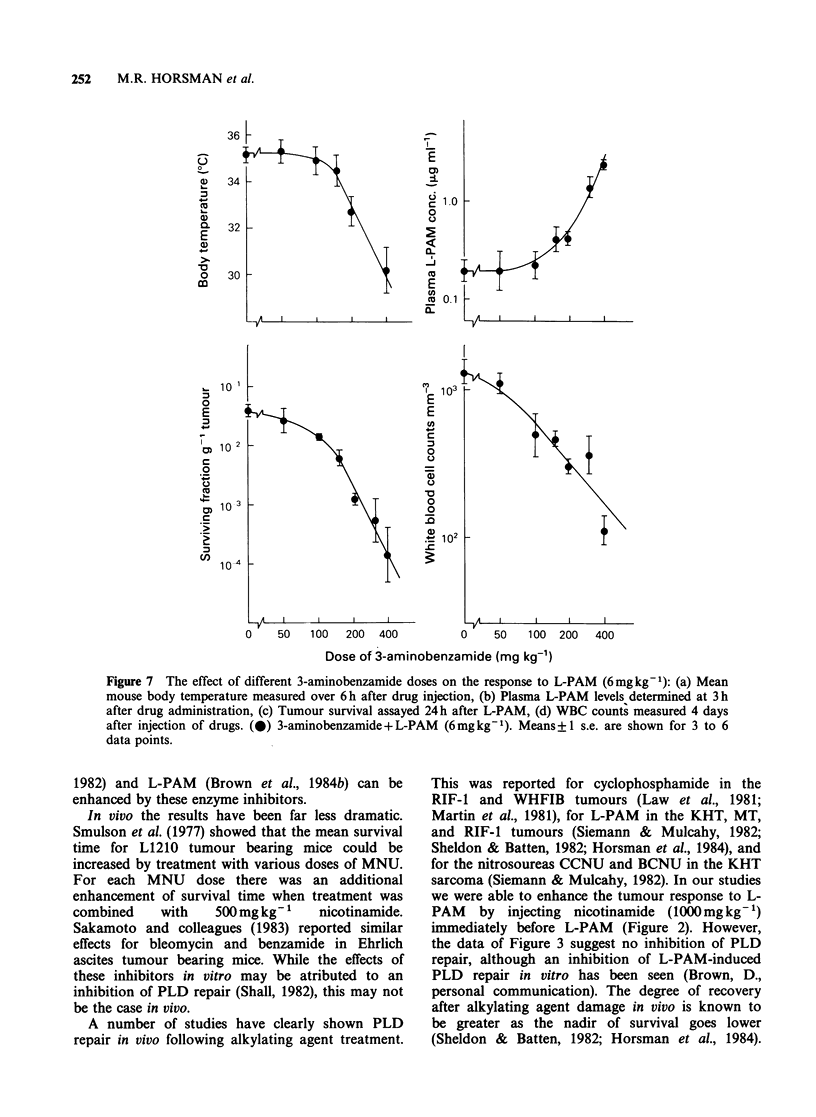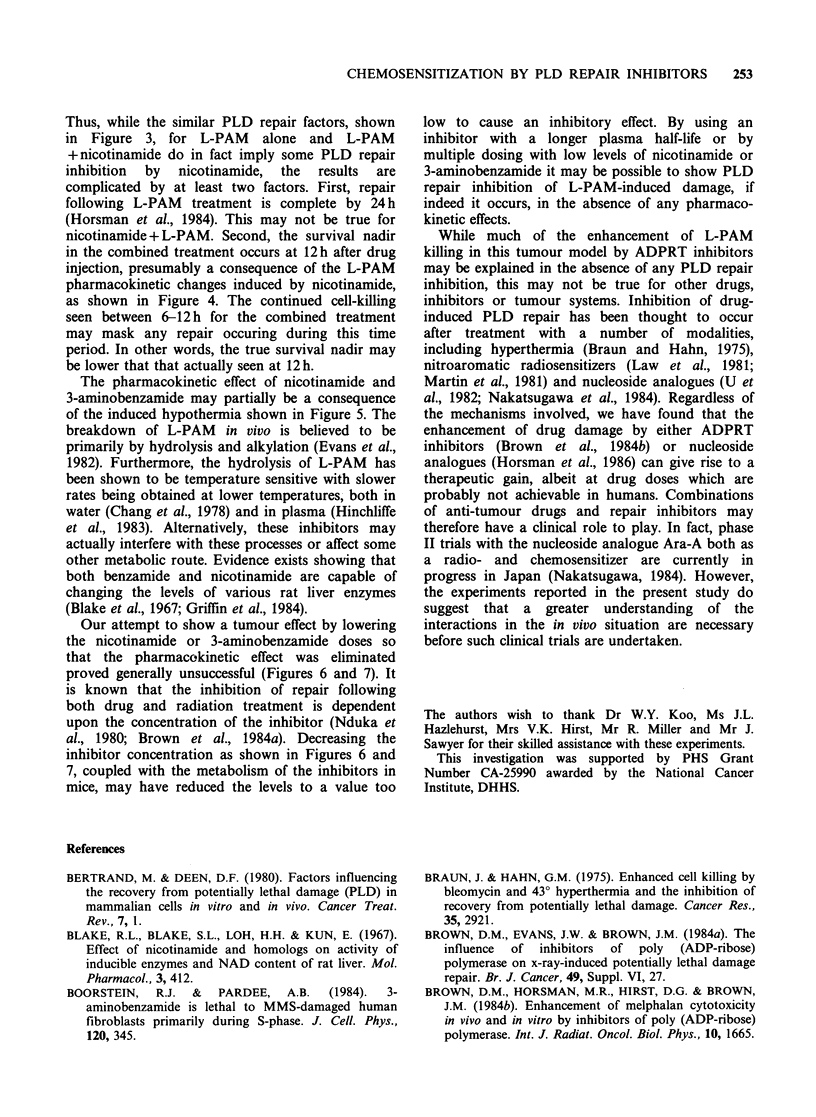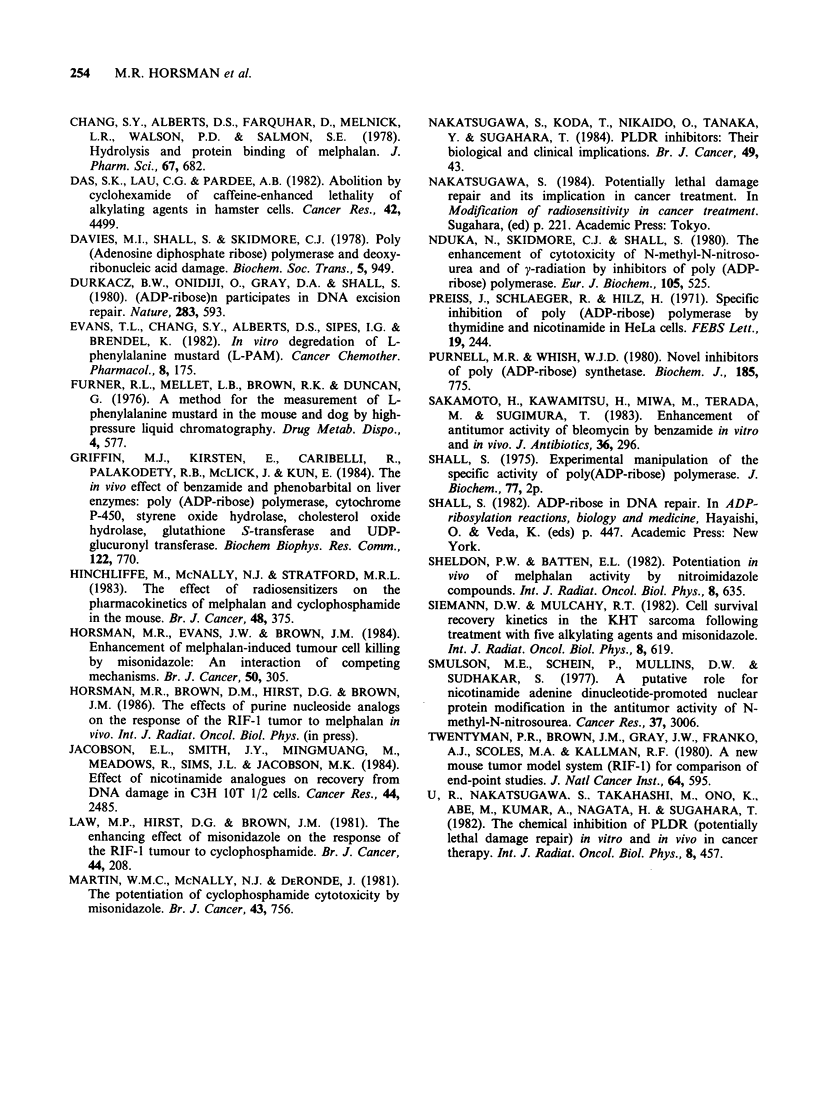# Changes in the response of the RIF-1 tumour to melphalan in vivo induced by inhibitors of nuclear ADP-ribosyl transferase.

**DOI:** 10.1038/bjc.1986.42

**Published:** 1986-02

**Authors:** M. R. Horsman, D. M. Brown, D. G. Hirst, J. M. Brown

## Abstract

The effect of inhibitors of nuclear ADP-ribosyl transferase (ADPRT) on the cytotoxicity of melphalan (L-PAM) in the RIF-1 tumour in vivo was investigated. A large single dose of nicotinamide (1000 mg kg-1) enhanced the tumour cell killing by L-PAM as measured by tumour cell survival. This enhancement was maximum when nicotinamide was administered within 1 h before injecting the L-PAM. When given at this time, the nicotinamide had a dose-modifying effect on all L-PAM doses tested, giving rise to a mean enhancement ratio (ER) of 2.2. Nicotinamide did not appear to inhibit the recovery from L-PAM induced potentially lethal damage. L-PAM (6 mg kg-1) produced a transient drop in mouse body temperature. This effect was both increased and prolonged by nicotinamide. In addition the inhibitor also delayed the clearance of L-PAM from the plasma of C3H mice, such that the half-life of the chemotherapeutic agent was extended from 41 min to 143 min. The effect of combining L-PAM with nicotinamide doses below 1000 mg kg-1 was also investigated. The results showed that as the nicotinamide dose was decreased, the enhancement of the effects on body temperature, pharmacokinetics and white blood cell counts were reduced. However, a concomitant loss in the enhancement of tumour cell killing was also observed. Similar results were obtained using 3-aminobenzamide, a more efficient inhibitor of ADPRT.


*Br. J. Cancer* (1986), **53**, 247–254

### Changes in the response of the RIF-1 tumour to melphalan *in vivo* induced by inhibitors of nuclear ADP-ribosyl transferase

M.R. Horsman, D.M. Brown, D.G. Hirst & J.M. Brown

*Division of Radiobiology Research, Department of Radiology, Stanford University, Stanford, CA 94305, USA.*

**Summary** The effect of inhibitors of nuclear ADP-ribosyl transferase (ADPRT) on the cytotoxicity of melphalan (L-PAM) in the RIF-1 tumour *in vivo* was investigated. A large single dose of nicotinamide ($1000\,mg\,kg^{-1}$) enhanced the tumour cell killing by L-PAM as measured by tumour cell survival. This enhancement was maximum when nicotinamide was administered within 1 h before injecting the L-PAM. When given at this time, the nicotinamide had a dose-modifying effect on all L-PAM doses tested, giving rise to a mean enhancement ratio (ER) of 2.2. Nicotinamide did not appear to inhibit the recovery from L-PAM induced potentially lethal damage. L-PAM ($6\,mg\,kg^{-1}$) produced a transient drop in mouse body temperature. This effect was both increased and prolonged by nicotinamide. In addition the inhibitor also delayed the clearance of L-PAM from the plasma of C3H mice, such that the half-life of the chemotherapeutic agent was extended from 41 min to 143 min. The effect of combining L-PAM with nicotinamide doses below $1000\,mg\,kg^{-1}$ was also investigated. The results showed that as the nicotinamide dose was decreased, the enhancement of the effects on body temperature, pharmacokinetics and white blood cell counts were reduced. However, a concomitant loss in the enhancement of tumour cell killing was also observed. Similar results were obtained using 3-aminobenzamide, a more efficient inhibitor of ADPRT.

A major factor affecting the response of malignant tumours to various chemotherapeutic agents may be their ability to repair potentially lethal damage (PLD). Recovery from PLD has been observed in tumour cells after drug treatment both *in vitro* and *in vivo* (for review see Bertrand & Deen, 1980). It remains a possibility that if this repair can be inhibited a better clinical response following drug therapy may be achieved.

There is now strong *in vitro* evidence that the enzyme, nuclear ADP-ribosyl transferase (ADPRT), participates in DNA excision repair after exposure of eukaryotic cells to radiation or alkylating agents, although the precise molecular involement of the enzyme is not yet known (for review see Shall, 1982). The activity of this enzyme can be inhibited by several groups of compounds including thymidine, nicotinamides, benzamides and methylxanthines (Preiss *et al.*, 1971; Shall, 1975; Davies *et al.*, 1978). Combining these inhibitors with DNA damaging agents has been shown to produce a significantly greater degree of cell killing than seen with these agents in the absence of the inhibitor. This has been reported for the enhancement of drug-induced damage *in vitro* (Nduka *et al.*, 1980; Jacobson *et al.*, 1984) and to a lesser extent *in vivo* (Smulson *et al.*, 1977; Sakamoto *et al.*, 1983).

In order to investigate these effects in more detail, we have studied the effect of two inhibitors of ADPRT, nicotinamide and 3-aminobenzamide, on the activity of the bifunctional alkylating agent melphalan (L-phenylalanine mustard, L-PAM) against normal and malignant tissues in the mouse. A preliminary report of this work has been presented (Brown *et al.*, 1984*b*).

### Materials and methods

### *Tumour system*

The RIF-1 tumour was used in all experiments. This tumor is a radiation-induced sarcoma which arose in the inbred female C3H/Km mouse. It is routinely maintained by passage *in vivo* and *in vitro* according to a published protocol (Twentyman *et al.*, 1980). Solid tumours were produced in 3–4 month old female C3H/Km mice by inoculating $2\times10^5$ cells into the gastrocnemius muscle in the right rear leg. All drug treatments were carried out when the tumour size was 300–600 mg. The experimental techniques have previously been described in detail (Horsman *et al.*, 1984).

Correspondence: M.R. Horsman.
Received 1 August 1985; and in revised form, 12 October 1985.

© The Macmillan Press, Ltd., 1986

*Drug treatments*

All drug solutions were prepared immediately prior to injection. Nicotinamide (Sigma Chemical Co., St. Louis, MO) was dissolved in a sterile saline solution (0.9% NaCl). 3-aminobenzamide (Sigma) was prepared by dissolving 100 mg in 1 ml absolute ethanol and diluting in 9 ml saline. Subsequent dilutions were in saline. L-PAM (Burroughs Wellcome Co., Research Triangle Park, NC) was prepared by dissolving 10 mg in 1 ml 95% ethanol and 5% HCl. It was further diluted to the required concentration in a 60% solution of propylene glycol in saline. Prepared drug concentrations were varied so that a constant volume (0.01 ml $g^{-1}$ body weight) could be injected for both nicotinamide and L-PAM. The low solubility of 3-aminobenzamide, however, necessitated the injection of 0.01 to 0.04 ml $g^{-1}$ body weight depending on the required final drug concentration. All drugs were injected i.p.

*Tumour studies*

Tumour response was assayed by survival of tumour cells. Survival was determined, as previously described (Horsman *et al.*, 1984), by excising tumours at various times, up to and including 24 h, after injecting L-PAM. Three tumours were combined for each datum point. They were subsequently minced, enzymatically disaggregated and single cell suspensions produced. The enzyme mixture was removed by centrifugation (1500 r.p.m.; 10 min) and the cells resuspended, counted, serially diluted and plated in Waymouth's medium + 15% foetal calf serum (Gibco, Santa Clara, CA). The colony-forming ability of these cells was then determined. Survival was expressed as surviving fraction $g^{-1}$ tumour. This is the product of the plating efficiency and cell yield $g^{-1}$ of treated tumours relative to that for untreated tumours.

*Normal tissue studies*

Previous experiments have shown that the number of white blood cells (WBC) in the peripheral blood declines for several days after treatment with cyclophosphamide (Law *et al.*, 1981) and L-PAM (Hirst, unpublished) reaching a minimum at ~4 days before recovery begins. In our experiments 5 $\mu$l blood samples were taken from the tails of tumour-bearing mice four days after injecting drugs. The blood was diluted with 95 $\mu$l of 2% glacial acetic acid to lyze the erythrocytes. The resulting suspension of leucocytes was counted using a haemacytometer. Six mice were used for each treatment group.

*Measurement of plasma L-PAM levels*

Plasma L-PAM levels were determined as described previously (Horsman *et al.*, 1984). Briefly, at various times after drug injection, mice were bled by cardiac puncture under diethyl ether anaesthesia and plasma separated by centrifugation (3,000 r.p.m.; 5 min). Drug concentrations were determined by reverse phase high-performance liquid chromatography (Waters Associates, Milford, MA) using the procedure of Furner *et al.* (1976). Results were collected on a data module chart recorder (Waters Associates). Quantitation of drug concentration was by peak area with reference to linear calibration curves.

*Body temperature measurements*

For the RIF-1 tumour grown i.m. in the leg of C3H mice, body temperatures are a good indicator of tumour temperatures (Horsman *et al.*, 1984). Drug-induced temperature changes were therefore determined by measuring mouse body temperatures at various times after drug injections using a rectally inserted thermocouple (Bailey Instruments, Saddle Brooke, NJ).

### Results

When tumour-bearing C3H mice were given nicotinamide (1000 mg $kg^{-1}$) and L-PAM (6 mg $kg^{-1}$) in combination, there was an enhancement of cell killing above that obtained with L-PAM alone (Figure 1). The effect was maximal when nicotinamide was given between 1 h before L-PAM and at the same time as the alkylating agent. This enhancement was reduced as the time interval between the drugs increased. Nicotinamide alone caused a small amount of cell killing. As a consequence of the results shown in Figure 1, all further experiments were carried out with the nicotinamide being administered immediately before the L-PAM.

The effect of this large single dose of nicotinamide on the L-PAM dose-response curve, as measured by tumour cell survival at 24 h after drug treatment, is shown in Figure 2. Increasing doses of L-PAM caused increasing amounts of cell kill. Nicotinamide alone had a small effect on the tumour response. However, when combined with L-PAM it significantly enhanced the response to the alkylating agent as demonstrated by a steeper dose response curve for the two agents combined. The data shown in Figure 2 suggests that nicotinamide had a dose-modifying effect for all L-PAM doses, giving a mean enhancement ratio (ER: calculated as ratio of drug concentration for L-PAM alone to

**Figure 1** The effect of drug timing on tumour cell survival. Nicotinamide ($1000\,mg\,kg^{-1}$) was injected at various times before or after L-PAM ($6\,mg\,kg^{-1}$) and survival of RIF-1 tumour cells assayed 24 h later: (○) nicotinamide + L-PAM diluent; (●) nicotinamide + L-PAM; The shaded area represents L-PAM only. Means ± 1 s.e. are shown for 3 to 4 data points.

**Figure 2** The effect of L-PAM ± nicotinamide ($1000\,mg\,kg^{-1}$) in the RIF-1 tumour as measured by tumour survival at 24 h after injection: (○) saline + L-PAM; (●) nicotinamide + L-PAM. Means ± 1 s.e. are shown for 4 separate experiments.

that obtained with L-PAM and nicotinamide to give the same response) of 2.2 (corrected for nicotinamide toxicity).

Figure 3 shows the effect on survival of varying the time of removal of tumours from the animals following an L-PAM injection. When mice were given saline immediately before a single injection of L-PAM ($6\,mg\,kg^{-1}$), significant toxicity was seen even when the tumours were removed within 2 h following the L-PAM dose. The nadir in survival was reached at 4 h and was followed by repair of L-PAM-induced potentially lethal damage (PLD), shown by an increasing surviving fraction from 4 to 24 h. Nicotinamide alone ($1000\,mg\,kg^{-1}$) reduced survival to ~60% over the 24 h period. In combination with L-PAM, it not only enhanced the degree of cell killing, but also moved the nadir to 12 h. After this time cell survival increased by about the same amount as seen with L-PAM alone, although the final survival level was between $1\frac{1}{2}$ to 2 decades lower.

**Figure 3** Survival of RIF-1 tumour cells as a function of time between drug administration and tumour removal: (▲) nicotinamide ($1000\,mg\,kg^{-1}$) + L-PAM diluent; (○) saline + L-PAM ($6\,mg\,kg^{-1}$); (●) nicotinamide ($1000\,mg\,kg^{-1}$) + L-PAM ($6\,mg\,kg^{-1}$). Means ± 1 s.e. for 4 separate experiments are shown.

Figure 4 shows plasma levels for L-PAM as a function of time after drug injections. Nicotinamide ($1000\,mg\,kg^{-1}$) given immediately before a dose of $6\,mg\,kg^{-1}$ L-PAM produced a marked slowing in the elimination of L-PAM from the blood. The half-

**Figure 4** Plasma levels of L-PAM in tumour-bearing C3H mice as a function of time after L-PAM administration: (○) saline + L-PAM (6 mg kg$^{-1}$); (●) nicotinamide (1000 mg kg$^{-1}$) + L-PAM (6 mg kg$^{-1}$). Each point represents the plasma level from one mouse, with the results from 3 separate experiments shown. Lines determined by linear regression analysis in the regions where exponential decays operated.

**Figure 5** Mouse body temperature as a function of time after drug administration: (△) saline + L-PAM diluent; (□) nicotinamide (1000 mg kg$^{-1}$); (○) nicotinamide (1000 mg kg$^{-1}$) + L-PAM diluent; (▲) saline + L-PAM (6 mg kg$^{-1}$); (●) nicotinamide (1000 mg kg$^{-1}$) + L-PAM (6 mg kg$^{-1}$). The shaded area represents the body temperature of untreated mice. Means ± 1 s.e. for 3 to 6 mice are shown.

life of L-PAM (±1 s.e.), in the region where the exponential decay operated, was extended from 41 min (31–59) to 143 min (117–185). Such a change in L-PAM pharmacokinetics produces a 4-fold increase in the area under the drug concentration *versus* time curve (AUC).

A large single dose of L-PAM (6 mg kg$^{-1}$) caused a drop in rectal temperature of ~4°C in C3H mice (Figure 5). This lower temperature was reached within 1 h after the injection of L-PAM, but was followed by recovery such that by 5 h the body temperature had returned to normal. Part of this effect was a result of the L-PAM diluent, as previously demonstrated (Horsman *et al.*, 1984). The injection of nicotinamide alone (1000 mg kg$^{-1}$) produced an effect similar to that seen with L-PAM. However, when nicotinamide preceded L-PAM, the fall in body temperature was greater (7–8°C) and more prolonged: the body temperature had not returned to normal 8 h after giving the drugs.

In order to test whether an enhancement of L-PAM tumour cell killing could occur in the absence of any effect on L-PAM pharmacokinetics or body temperature, experiments were performed with reduced nicotinamide doses. As illustrated in Figure 6, when the nicotinamide dose injected immediately before a single dose of L-PAM (6 mg kg$^{-1}$) was decreased, there was a concomitant reduction in the fall in mean body temperature (measured over 6 h after drug injection) and the change in plasma levels of L-PAM (determined 3 h after injection). Below 125 mg kg$^{-1}$ nicotinamide these changes were virtually eliminated. This change in nicotinamide dose also produced a similar effect on the L-PAM-induced tumour cell killing, such that at the doses where no modification of body temperature or pharmacokinetics were observed, no significant enhancement of tumour response could be obtained. The effect of nicotinamide dose on a normal tissue is shown in Figure 6(d). White blood cell (WBC) counts were selected because haemotological effects are the major dose-limiting problem with alkylating agents. These counts were determined 4 days after injecting the drugs, when the WBC nadir is achieved following L-PAM administration (Hirst, unpublished). Any possible effect of nicotinamide dose on the subsequent recovery of WBC counts was not investigated. As shown in Figure 6(d), the L-PAM-induced

**Figure 6** The effect of different nicotinamide doses on the response to L-PAM ($6\,mg\,kg^{-1}$): (a) Mean mouse body temperature measured over 6 h after drug injection, (b) Plasma L-PAM levels determined at 3 h after drug administration, (c) Tumour survival assayed 24 h after L-PAM, (d) WBC counts measured 4 days after injection of drugs. (●) nicotinamide + L-PAM ($6\,mg\,kg^{-1}$). Means $\pm$ 1 s.e. are shown for 3 to 6 data points.

reduction in WBC counts were similarly affected by changes in nicotinamide dose as reported for the other endpoints shown in Figure 6.

Since 3-aminobenzamide is a more efficient inhibitor of ADPRT than nicotinamide (Purnell & Whish, 1980), we repeated these experiments with this compound. The results are shown in Figure 7. Although 3-aminobenzamide was more effective than nicotinamide on a concentration basis, its effects on all the end points studied were similar to those obtained with nicotinamide.

### Discussion

The present studies demonstrate that an enhancement of tumour response to the bifunctional alkylating agent L-PAM can be achieved with the ADPRT inhibitors nicotinamide and 3-aminobenzamide. Most of this effect appears to be the result of an increased plasma half-life of the chemotherapeutic agent and may not involve any inhibition of the repair of L-PAM-induced PLD.

Nduka *et al.* (1980) demonstrated that in L1210 cells the cytotoxicity of methylnitrosourea (MNU) could be potentiated if these cells were exposed to non-toxic concentrations of various ADPRT inhibitors during exposure to the cytotoxic agent and the subsequent cloning period. Similar results have been reported for dimethyl sulphate (Durkacz *et al.*, 1980), streptozotocin (Shall, 1982), methyl methane sulphonate (Boorstein & Pardee, 1984) and for recovery from N-methyl-N′-nitro-N-nitrosoguanidine damage (Jacobson *et al.*, 1984). In addition, the *in vitro* cytotoxicity of the bifunctional alkylating agents nitrogen mustard (Das *et al.*,

**Figure 7** The effect of different 3-aminobenzamide doses on the response to L-PAM ($6\,mg\,kg^{-1}$): (a) Mean mouse body temperature measured over 6 h after drug injection, (b) Plasma L-PAM levels determined at 3 h after drug administration, (c) Tumour survival assayed 24 h after L-PAM, (d) WBC counts measured 4 days after injection of drugs. (●) 3-aminobenzamide + L-PAM ($6\,mg\,kg^{-1}$). Means $\pm 1$ s.e. are shown for 3 to 6 data points.

1982) and L-PAM (Brown *et al.*, 1984*b*) can be enhanced by these enzyme inhibitors.

*In vivo* the results have been far less dramatic. Smulson *et al.* (1977) showed that the mean survival time for L1210 tumour bearing mice could be increased by treatment with various doses of MNU. For each MNU dose there was an additional enhancement of survival time when treatment was combined with $500\,mg\,kg^{-1}$ nicotinamide. Sakamoto and colleagues (1983) reported similar effects for bleomycin and benzamide in Ehrlich ascites tumour bearing mice. While the effects of these inhibitors *in vitro* may be atributed to an inhibition of PLD repair (Shall, 1982), this may not be the case *in vivo*.

A number of studies have clearly shown PLD repair *in vivo* following alkylating agent treatment. This was reported for cyclophosphamide in the RIF-1 and WHFIB tumours (Law *et al.*, 1981; Martin *et al.*, 1981), for L-PAM in the KHT, MT, and RIF-1 tumours (Siemann & Mulcahy, 1982; Sheldon & Batten, 1982; Horsman *et al.*, 1984), and for the nitrosoureas CCNU and BCNU in the KHT sarcoma (Siemann & Mulcahy, 1982). In our studies we were able to enhance the tumour response to L-PAM by injecting nicotinamide ($1000\,mg\,kg^{-1}$) immediately before L-PAM (Figure 2). However, the data of Figure 3 suggest no inhibition of PLD repair, although an inhibition of L-PAM-induced PLD repair *in vitro* has been seen (Brown, D., personal communication). The degree of recovery after alkylating agent damage *in vivo* is known to be greater as the nadir of survival goes lower (Sheldon & Batten, 1982; Horsman *et al.*, 1984).

Thus, while the similar PLD repair factors, shown in Figure 3, for L-PAM alone and L-PAM +nicotinamide do in fact imply some PLD repair inhibition by nicotinamide, the results are complicated by at least two factors. First, repair following L-PAM treatment is complete by 24h (Horsman *et al.*, 1984). This may not be true for nicotinamide+L-PAM. Second, the survival nadir in the combined treatment occurs at 12h after drug injection, presumably a consequence of the L-PAM pharmacokinetic changes induced by nicotinamide, as shown in Figure 4. The continued cell-killing seen between 6–12h for the combined treatment may mask any repair occuring during this time period. In other words, the true survival nadir may be lower that that actually seen at 12h.

The pharmacokinetic effect of nicotinamide and 3-aminobenzamide may partially be a consequence of the induced hypothermia shown in Figure 5. The breakdown of L-PAM *in vivo* is believed to be primarily by hydrolysis and alkylation (Evans *et al.*, 1982). Furthermore, the hydrolysis of L-PAM has been shown to be temperature sensitive with slower rates being obtained at lower temperatures, both in water (Chang *et al.*, 1978) and in plasma (Hinchliffe *et al.*, 1983). Alternatively, these inhibitors may actually interfere with these processes or affect some other metabolic route. Evidence exists showing that both benzamide and nicotinamide are capable of changing the levels of various rat liver enzymes (Blake *et al.*, 1967; Griffin *et al.*, 1984).

Our attempt to show a tumour effect by lowering the nicotinamide or 3-aminobenzamide doses so that the pharmacokinetic effect was eliminated proved generally unsuccessful (Figures 6 and 7). It is known that the inhibition of repair following both drug and radiation treatment is dependent upon the concentration of the inhibitor (Nduka *et al.*, 1980; Brown *et al.*, 1984*a*). Decreasing the inhibitor concentration as shown in Figures 6 and 7, coupled with the metabolism of the inhibitors in mice, may have reduced the levels to a value too low to cause an inhibitory effect. By using an inhibitor with a longer plasma half-life or by multiple dosing with low levels of nicotinamide or 3-aminobenzamide it may be possible to show PLD repair inhibition of L-PAM-induced damage, if indeed it occurs, in the absence of any pharmacokinetic effects.

While much of the enhancement of L-PAM killing in this tumour model by ADPRT inhibitors may be explained in the absence of any PLD repair inhibition, this may not be true for other drugs, inhibitors or tumour systems. Inhibition of drug-induced PLD repair has been thought to occur after treatment with a number of modalities, including hyperthermia (Braun and Hahn, 1975), nitroaromatic radiosensitizers (Law *et al.*, 1981; Martin *et al.*, 1981) and nucleoside analogues (U *et al.*, 1982; Nakatsugawa *et al.*, 1984). Regardless of the mechanisms involved, we have found that the enhancement of drug damage by either ADPRT inhibitors (Brown *et al.*, 1984*b*) or nucleoside analogues (Horsman *et al.*, 1986) can give rise to a therapeutic gain, albeit at drug doses which are probably not achievable in humans. Combinations of anti-tumour drugs and repair inhibitors may therefore have a clinical role to play. In fact, phase II trials with the nucleoside analogue Ara-A both as a radio- and chemosensitizer are currently in progress in Japan (Nakatsugawa, 1984). However, the experiments reported in the present study do suggest that a greater understanding of the interactions in the *in vivo* situation are necessary before such clinical trials are undertaken.

The authors wish to thank Dr W.Y. Koo, Ms J.L. Hazlehurst, Mrs V.K. Hirst, Mr R. Miller and Mr J. Sawyer for their skilled assistance with these experiments.

This investigation was supported by PHS Grant Number CA-25990 awarded by the National Cancer Institute, DHHS.

### References

BERTRAND, M. & DEEN, D.F. (1980). Factors influencing the recovery from potentially lethal damage (PLD) in mammalian cells *in vitro* and *in vivo*. *Cancer Treat. Rev.*, **7**, 1.

BLAKE, R.L., BLAKE, S.L., LOH, H.H. & KUN, E. (1967). Effect of nicotinamide and homologs on activity of inducible enzymes and NAD content of rat liver. *Mol. Pharmacol.*, **3**, 412.

BOORSTEIN, R.J. & PARDEE, A.B. (1984). 3-aminobenzamide is lethal to MMS-damaged human fibroblasts primarily during S-phase. *J. Cell. Phys.*, **120**, 345.

BRAUN, J. & HAHN, G.M. (1975). Enhanced cell killing by bleomycin and 43° hyperthermia and the inhibition of recovery from potentially lethal damage. *Cancer Res.*, **35**, 2921.

BROWN, D.M., EVANS, J.W. & BROWN, J.M. (1984*a*). The influence of inhibitors of poly (ADP-ribose) polymerase on x-ray-induced potentially lethal damage repair. *Br. J. Cancer*, **49**, Suppl. VI, 27.

BROWN, D.M., HORSMAN, M.R., HIRST, D.G. & BROWN, J.M. (1984*b*). Enhancement of melphalan cytotoxicity *in vivo* and *in vitro* by inhibitors of poly (ADP-ribose) polymerase. *Int. J. Radiat. Oncol. Biol. Phys.*, **10**, 1665.

CHANG, S.Y., ALBERTS, D.S., FARQUHAR, D., MELNICK, L.R., WALSON, P.D. & SALMON, S.E. (1978). Hydrolysis and protein binding of melphalan. *J. Pharm. Sci.*, **67**, 682.

DAS, S.K., LAU, C.G. & PARDEE, A.B. (1982). Abolition by cyclohexamide of caffeine-enhanced lethality of alkylating agents in hamster cells. *Cancer Res.*, **42**, 4499.

DAVIES, M.I., SHALL, S. & SKIDMORE, C.J. (1978). Poly (Adenosine diphosphate ribose) polymerase and deoxyribonucleic acid damage. *Biochem. Soc. Trans.*, **5**, 949.

DURKACZ, B.W., ONIDIJI, O., GRAY, D.A. & SHALL, S. (1980). (ADP-ribose)n participates in DNA excision repair. *Nature*, **283**, 593.

EVANS, T.L., CHANG, S.Y., ALBERTS, D.S., SIPES, I.G. & BRENDEL, K. (1982). *In vitro* degredation of L-phenylalanine mustard (L-PAM). *Cancer Chemother. Pharmacol.*, **8**, 175.

FURNER, R.L., MELLET, L.B., BROWN, R.K. & DUNCAN, G. (1976). A method for the measurement of L-phenylalanine mustard in the mouse and dog by high-pressure liquid chromatography. *Drug Metab. Dispo.*, **4**, 577.

GRIFFIN, M.J., KIRSTEN, E., CARIBELLI, R., PALAKODETY, R.B., McLICK, J. & KUN, E. (1984). The *in vivo* effect of benzamide and phenobarbital on liver enzymes: poly (ADP-ribose) polymerase, cytochrome P-450, styrene oxide hydrolase, cholesterol oxide hydrolase, glutathione *S*-transferase and UDP-glucuronyl transferase. *Biochem Biophys. Res. Comm.*, **122**, 770.

HINCHLIFFE, M., McNALLY, N.J. & STRATFORD, M.R.L. (1983). The effect of radiosensitizers on the pharmacokinetics of melphalan and cyclophosphamide in the mouse. *Br. J. Cancer*, **48**, 375.

HORSMAN, M.R., EVANS, J.W. & BROWN, J.M. (1984). Enhancement of melphalan-induced tumour cell killing by misonidazole: An interaction of competing mechanisms. *Br. J. Cancer*, **50**, 305.

HORSMAN, M.R., BROWN, D.M., HIRST, D.G. & BROWN, J.M. (1986). The effects of purine nucleoside analogs on the response of the RIF-1 tumor to melphalan *in vivo*. *Int. J. Radiat. Oncol. Biol. Phys.* (in press).

JACOBSON, E.L., SMITH, J.Y., MINGMUANG, M., MEADOWS, R., SIMS, J.L. & JACOBSON, M.K. (1984). Effect of nicotinamide analogues on recovery from DNA damage in C3H 10T 1/2 cells. *Cancer Res.*, **44**, 2485.

LAW, M.P., HIRST, D.G. & BROWN, J.M. (1981). The enhancing effect of misonidazole on the response of the RIF-1 tumour to cyclophosphamide. *Br. J. Cancer*, **44**, 208.

MARTIN, W.M.C., McNALLY, N.J. & DeRONDE, J. (1981). The potentiation of cyclophosphamide cytotoxicity by misonidazole. *Br. J. Cancer*, **43**, 756.

NAKATSUGAWA, S., KODA, T., NIKAIDO, O., TANAKA, Y. & SUGAHARA, T. (1984). PLDR inhibitors: Their biological and clinical implications. *Br. J. Cancer*, **49**, 43.

NAKATSUGAWA, S. (1984). Potentially lethal damage repair and its implication in cancer treatment. In *Modification of radiosensitivity in cancer treatment*. Sugahara, (ed) p. 221. Academic Press: Tokyo.

NDUKA, N., SKIDMORE, C.J. & SHALL, S. (1980). The enhancement of cytotoxicity of N-methyl-N-nitrosourea and of $\gamma$-radiation by inhibitors of poly (ADP-ribose) polymerase. *Eur. J. Biochem.*, **105**, 525.

PREISS, J., SCHLAEGER, R. & HILZ, H. (1971). Specific inhibition of poly (ADP-ribose) polymerase by thymidine and nicotinamide in HeLa cells. *FEBS Lett.*, **19**, 244.

PURNELL, M.R. & WHISH, W.J.D. (1980). Novel inhibitors of poly (ADP-ribose) synthetase. *Biochem. J.*, **185**, 775.

SAKAMOTO, H., KAWAMITSU, H., MIWA, M., TERADA, M. & SUGIMURA, T. (1983). Enhancement of antitumor activity of bleomycin by benzamide *in vitro* and *in vivo*. *J. Antibiotics*, **36**, 296.

SHALL, S. (1975). Experimental manipulation of the specific activity of poly(ADP-ribose) polymerase. *J. Biochem.*, **77**, 2p.

SHALL, S. (1982). ADP-ribose in DNA repair. In *ADP-ribosylation reactions, biology and medicine*, Hayaishi, O. & Veda, K. (eds) p. 447. Academic Press: New York.

SHELDON, P.W. & BATTEN, E.L. (1982). Potentiation *in vivo* of melphalan activity by nitroimidazole compounds. *Int. J. Radiat. Oncol. Biol. Phys.*, **8**, 635.

SIEMANN, D.W. & MULCAHY, R.T. (1982). Cell survival recovery kinetics in the KHT sarcoma following treatment with five alkylating agents and misonidazole. *Int. J. Radiat. Oncol. Biol. Phys.*, **8**, 619.

SMULSON, M.E., SCHEIN, P., MULLINS, D.W. & SUDHAKAR, S. (1977). A putative role for nicotinamide adenine dinucleotide-promoted nuclear protein modification in the antitumor activity of N-methyl-N-nitrosourea. *Cancer Res.*, **37**, 3006.

TWENTYMAN, P.R., BROWN, J.M., GRAY, J.W., FRANKO, A.J., SCOLES, M.A. & KALLMAN, R.F. (1980). A new mouse tumor model system (RIF-1) for comparison of end-point studies. *J. Natl Cancer Inst.*, **64**, 595.

U, R., NAKATSUGAWA, S., TAKAHASHI, M., ONO, K., ABE, M., KUMAR, A., NAGATA, H. & SUGAHARA, T. (1982). The chemical inhibition of PLDR (potentially lethal damage repair) *in vitro* and *in vivo* in cancer therapy. *Int. J. Radiat. Oncol. Biol. Phys.*, **8**, 457.